# Real-Time Volatile Metabolomics Analysis of Dendritic Cells

**DOI:** 10.1021/acs.analchem.3c00516

**Published:** 2023-06-13

**Authors:** Kim Arnold, Philippe Dehio, Jonas Lötscher, Kapil Dev Singh, Diego García-Gómez, Christoph Hess, Pablo Sinues, Maria L. Balmer

**Affiliations:** † University Children’s Hospital Basel (UKBB), 4056 Basel, Switzerland; ‡ Department of Biomedical Engineering, University of Basel, 4123 Allschwil, Switzerland; § Department of Biomedicine, Immunobiology, University of Basel and University Hospital of Basel, 4031 Basel, Switzerland; ∥ Department of Analytical Chemistry, Nutrition and Food Science, University of Salamanca, 37008 Salamanca, Spain; ⊥ Department of Medicine, CITIID, Jeffrey Cheah Biomedical Centre, University of Cambridge, Cambridge CB2 0AW, United Kingdom; # Department of Biomedical Research (DBMR), University of Bern, 3008 Bern, Switzerland; ○ University Clinic for Diabetes, Endocrinology, Clinical Nutrition and Metabolism, Inselspital, 3010 Bern, Switzerland; □ Diabetes Center Bern (DCB), 3010 Bern, Switzerland

## Abstract

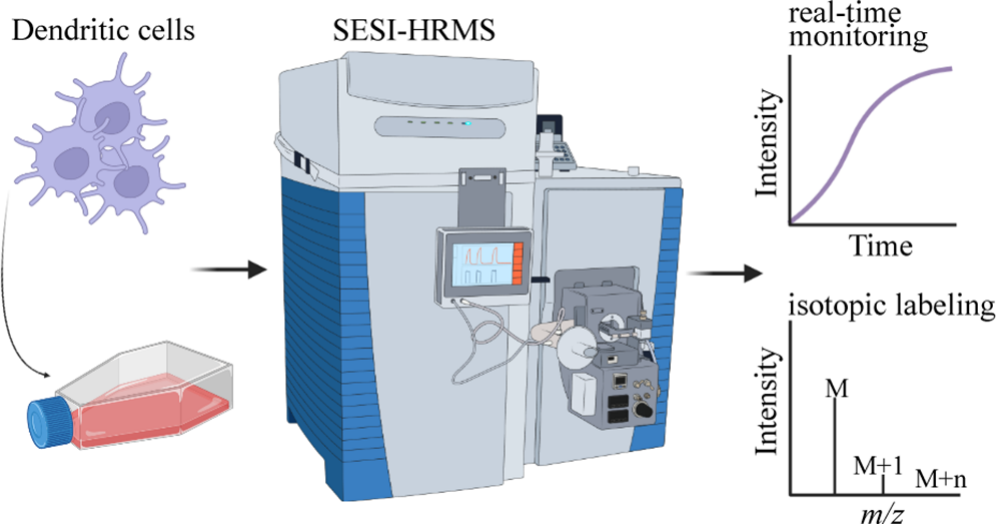

Dendritic cells (DCs) actively sample and present antigen to cells of the adaptive immune system and are thus vital for successful immune control and memory formation. Immune cell metabolism and function are tightly interlinked, and a better understanding of this interaction offers potential to develop immunomodulatory strategies. However, current approaches for assessing the immune cell metabolome are often limited by end-point measurements, may involve laborious sample preparation, and may lack unbiased, temporal resolution of the metabolome. In this study, we present a novel setup coupled to a secondary electrospray ionization-high resolution mass spectrometric (SESI-HRMS) platform allowing headspace analysis of immature and activated DCs in real-time with minimal sample preparation and intervention, with high technical reproducibility and potential for automation. Distinct metabolic signatures of DCs treated with different supernatants (SNs) of bacterial cultures were detected during real-time analyses over 6 h compared to their respective controls (SN only). Furthermore, the technique allowed for the detection of ^13^C-incorporation into volatile metabolites, opening the possibility for real-time tracing of metabolic pathways in DCs. Moreover, differences in the metabolic profile of naïve and activated DCs were discovered, and pathway-enrichment analysis revealed three significantly altered pathways, including the TCA cycle, α-linolenic acid metabolism, and valine, leucine, and isoleucine degradation.

## Introduction

With the recent rise of metabolomics technologies, research on immunometabolism has boomed during the past years, especially due to findings that metabolic reprogramming is essential for cell maintenance, function, and differentiation.^1,2^ Characterizing and understanding cells at the metabolic level are relevant to better understand and potentially modify immune functionality in various diseases from infections to autoimmune diseases. A particular focus hereby may be given to dendritic cells (DCs), as they are the key mediators between the innate and adaptive immune system and their metabolic rewiring during development and activation is still incompletely understood.^3^

Currently, various techniques are deployed to assess cells’ immunometabolism depending on the hypothesis and cell system used.^1^ Usually, to get a first impression on metabolic alterations, quick and cost efficient measurements on nitric oxide (NO) consumption/arginase production or glucose consumption/lactate production can be combined with functional assays such as enzyme-linked immunosorbent assays (ELISA).^4^ For more in-depth metabolic characterization, real-time extracellular flux analysis (EFA) enables a parallel readout on glycolysis and oxidative phosphorylation (OXPHOS).^5^ However, the important limitations of all the aforementioned methods are that they mainly provide information on only a few parameters related to specific core pathways at a single time point (except EFA) or that they may affect the metabolic state of the cells when pretreatment of cells is required.^4^ To get a more detailed metabolic picture, previously described techniques can be combined with gold standard liquid chromatography–mass spectrometry (LC-MS) and gas chromatography–mass spectrometric (GC-MS) methods. Through targeted and untargeted LC-MS and GC-MS experiments, detailed information on metabolites of interest can be captured; however, those methods require time-consuming sample preparation and do not provide information on dynamic processes without using isotopically labeled substrates.^6,7^

Secondary electrospray ionization–high resolution mass spectrometry (SESI-HRMS) is an emerging technique and may be an attractive alternative metabolomics approach, as it can provide quick, sensitive, noninvasive, and real-time results with minimal sample preparation. SESI is a soft ionization method, operates at ambient pressure, and is able to detect volatile organic compounds (VOCs) with limits of detection in the low parts per trillion (ppt) range.^8^ When combined with HRMS it, in addition, allows the resolving of fine isotopic structures, which is suitable for isotopic tracing.^9^ Several mass spectrometric methods have been described in the literature to assess VOCs emitted from living organisms in enclosed controlled environments. These include bacteria,^10^ yeast,^11,12^ plants,^13^ mammalian cells,^14−16^ and small-animal models.^17^ Thus, the objective of the current study was to evaluate the potential of SESI-HRMS to assess DC metabolism as a model immune cell. Such a system could potentially open new possibilities for delivering valuable dynamic information on the crosstalk between immunity and cell metabolism.

## Experimental Section

The applicability of the new technical setup was subdivided into three studies. In study I we characterized VOCs emitted by DCs in real-time upon stimulation with two bacterial supernatants from two different time points (SN1 and SN2) of an *E. coli* culture. In study II, ^13^C-incorporation from labeled glucose (Glc ^13^C_6_) in VOCs emitted by DCs was monitored in real-time over 4 h. In study III, we assessed ^13^C-incorporation from Glc ^13^C_6_ in VOCs emitted by DCs after 24 h incubation with and without long time bacterial lipopolysaccharide (LPS) stimulation. In study I, bacterial SN was used as a surrogate stimulus for DC activation, since it contains a variety of bacterial structural components (PAMPs) as well as bacterial metabolites. SNs from two different time points were used in order to mimic different bacterial concentrations and growth rates. To narrow down the stimulation to toll-like receptor-agonists only, LPS was used in study III, as it is the best studied stimulus in the literature with a well-characterized metabolic response in DCs. In the following, the key experimental information, including sample preparation and the main analytical instrumentation, is described. Detailed information on SESI-HRMS data analysis, LC-MS/MS analysis performed in the case of compound identification, and flow cytometric viability analysis is provided in the Supporting Information.

### Cell Culture and Differentiation of Bone-Marrow-Derived Dendritic Cells (BMDCs)

Murine bone marrow for BMDC differentiation was isolated from the tibias and femurs of 5–10-week-old C57BL/6NCrl mice by flushing with complete medium (RPMI1640 containing 10% heat-inactivated fetal calf serum; FCS). Animals were kept in a specific pathogen-free facility. A total of 2 × 10^6^ bone marrow cells per Petri dish were incubated in the presence of 10 ng/mL GM-CSF for 8 days with a medium exchange on days 3 and 6. After 8 days, the floating cells were harvested and frozen in 10% DMSO and 90% FCS. On the days of the experiments, the cells were thawed, counted, and 5 × 10^6^ cells (unless indicated differently) were plated in a cell culture flask (Jet Biofil) and rested for 2 h at 37 °C preceding the measurement. For the measurements, four flasks with different conditions were simultaneously compared. For isotopic ^13^C glucose tracing, the medium was supplemented with dialyzed heat-inactivated FCS.

### Sample Preparation Study I

For experiments with bacterial SN, *E. coli* JM83 was incubated in LB-medium at 37 °C, shaken, and grown to an optical density (OD) of 0.4 (SN1) or 1.4 (SN2), centrifuged and sterile-filtered through a 0.22 μm filter. Two flasks contained DCs in 5 mL of medium, and the other two flasks contained 5 mL of medium only. A baseline headspace measurement was conducted for 1 h while switching between the four flasks every 5 min using the valve system. After the baseline measurement, sterile-filtered bacterial SN was diluted 1:4 in 5 mL of medium, which was then used to replace the medium used for baseline measurement in all four culture flasks. After the addition of the respective SN, real-time SESI-HRMS measurements were conducted in positive ionization mode for 6 h while switching between the four flasks every 5 min. This experiment was repeated on three different days with three different biological replicates/cell batches (*n* = 3).

### Sample Preparation Study II

To investigate the incorporation of ^13^C over time, three biological replicates/cell batches were used (*n* = 3); one batch each for two flasks, respectively. A total of 5 × 10^6^ cells were used for the batches DC1 and DC2, whereas 10^7^ cells were used for batch DC3. A baseline measurement of 40 min was conducted with cells resting in glucose-free medium. Afterward, Glc ^12^C_6_ or Glc ^13^C_6_ was dissolved in glucose-free medium to reach a concentration of 66 mM. One mL of the 66 mM solution was then added to each flask containing 5 mL of glucose-free medium to reach a final concentration of 11 mM, which is equal to the glucose concentration found in complete medium. For each of the three cell batches, one flask contained Glc ^12^C_6_ and the other contained Glc ^13^C_6_. After the addition of the glucose, real-time SESI-HRMS measurements were conducted in the positive ionization mode for 4 h, while switching between the flasks every 5 min.

### Sample Preparation Study III

To evaluate ^13^C-incorporation with and without long-time LPS stimulation, a total of 20 flasks with four different cell batches were prepared. Four flasks (*n* = 4) were always assigned to one of five groups, named G1 (Glc ^12^C_6_ in medium + LPS), G2 (Glc ^12^C_6_ in medium + DC), G3 (Glc ^12^C_6_ in medium + DC + LPS), G4 (Glc ^13^C_6_ in medium + DC), and G5 (Glc ^13^C_6_ in medium + DC + LPS), respectively. The glucose concentrations were the same as described in study II. All flasks were incubated for 24–27 h at 37 °C, 5% CO_2_. After incubation, the four flasks of each group were sequentially measured by SESI-HRMS for 5 min each. The experiment was conducted in positive and negative ionization mode.

### Real-Time Cell Culture Headspace Measurement with SESI-HRMS

**Figure 1:**
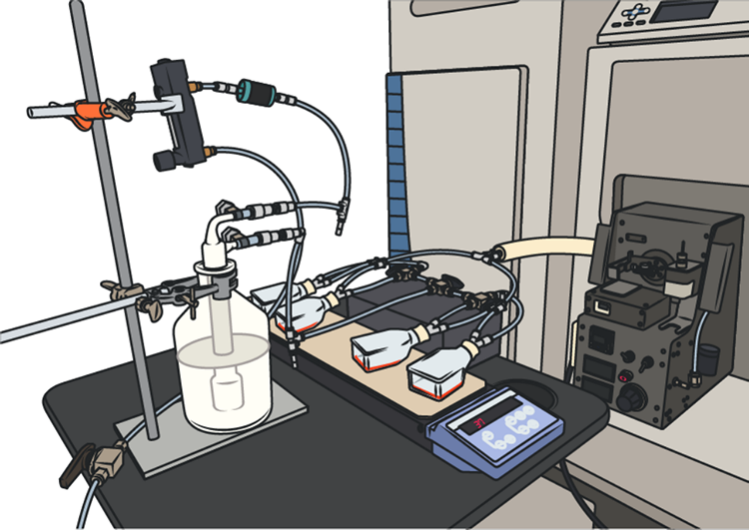
Experimental setup for DC headspace measurement by SESI-HRMS. Cell culture flasks were simultaneously attached to the SESI-HRMS via PTFE tubes and kept at 37 °C using a multipoint heating plate. Integrated 3-way ball valves allowed switching between the samples. The system was pervaded with a humidified gas mixture at a flow rate of 0.3 L/min to carry VOCs emitted by DCs toward the SESI-HRMS for real-time analysis.

A scheme of the general experimental setup is shown in Figure 1. Briefly, the setup consisted of four cell-culture flasks sealed airtight with inert rubber stoppers (HUBERLAB, Aesch) containing two holes for introducing polytetrafluoroethylene (PTFE) tubes. Stainless steel 3-way ball valves (Swagelok) enabled alternating switching between the four flasks simultaneously, connected via PTFE tubes to the SESI-HRMS. The flasks were placed on a multipoint heating plate (Carl Roth, Arlesheim) and were kept at a temperature of 37 °C during the experiments. A gas mixture (5% CO_2_, 16% O_2_, and 79% N_2_) was used to pervade the system at a flow rate of 0.3 L/min and carried the VOCs emitted by the respective flasks’ contents toward the SESI-HRMS. To prevent the contents in the flasks from drying out, a bottle filled with LC-MS-grade water was built into the system to ensure a humid environment. Usually, a time-span of 5 min was needed to measure a single flask. To ensure reproducibility of measurements on different days, a quality control standard gas mixture was infused 1 h before the start of each experiment. The protocol followed for quality control measurements is detailed by Gisler et al.^18^

### SESI-HRMS Analytical Platform

The analytical platform consisted of an ion source (Super SESI, FIT, Spain) coupled to a high-resolution mass spectrometer (Q-Exactive Plus, Thermo Fisher Scientific, Germany). Mass spectra were acquired via Thermo Exactive Plus Tune software (version 2.9) in full scan mode (scan range 50–400 *m*/*z*, polarity positive or negative, microscan number 10, ACG target 106, and maximum injection time 500 ms) at a resolution of 280000 at *m*/*z* 200. For the formation of the electrospray, 20 μm ID TaperTip silica capillary emitter (New Objective, U.S.A.) was used in study I, and a 20 μm ID nanoelectrospray capillary (Fossil Ion Tech, Spain) was used in studies II and III along with 0.1% formic acid in water. Pressure of the SESI solvent environment was set to 1.3 bar (study I) and 0.8 bar (studies II and III). The electrospray voltage was set to 3.5 kV in positive and 2.8 kV in negative ionization mode. The temperatures of the ionization chamber and sampling line were set to 90 and 130 °C, respectively. Capillary temperature was 275 °C, sheath gas was set to 60, and S-lens RF level was set to 55.0. The mass flow controller exhaust set point was 0.7 L/min, and a nitrogen stream through the source was set to 0.4 L/min to ensure a constant flow of the headspace gas to the ionizer (0.3 L/min). In addition, the system was calibrated weekly internally and externally using common background contaminant lock masses and room air.^19,20^

## Results and Discussion

### Study I: Stimulation of Cells by Bacterial SN

**Figure 2:**
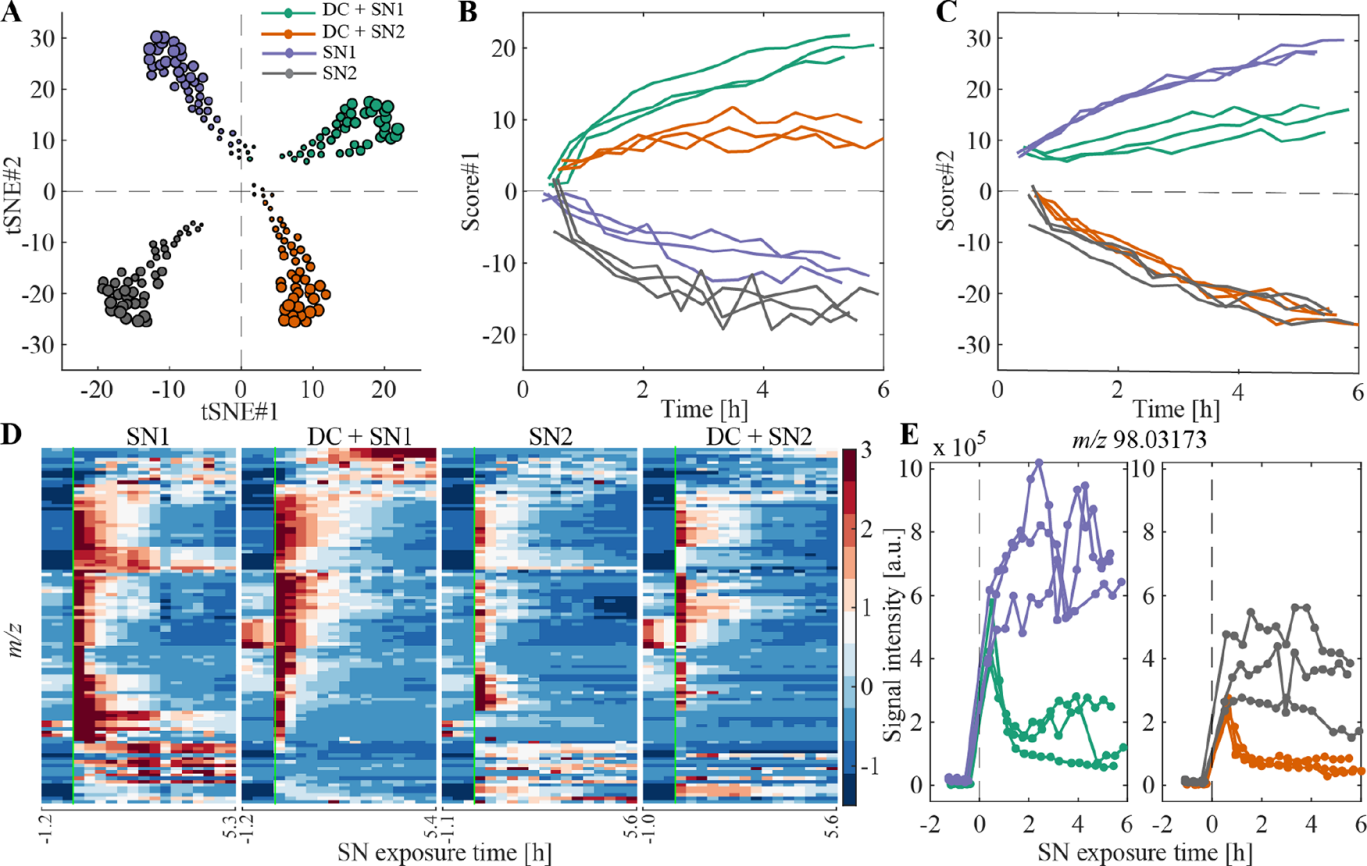
Distinguishing experimental conditions with high technical and biological reproducibility. (A) tSNE plot after SN addition of total 108 features which were significantly different (*p* ≤ 0.05) between DC + SN1 and SN1 or significantly different (*p* ≤ 0.05) between DC + SN2 and SN2 and showed a log 2FC ≥ 1 vs their respective baselines. The size of the symbols represents increasing time upon SN addition. (B) tSNE score 1 over time, separating samples containing DCs vs SN. (C) tSNE score 2 over time, separating samples containing SN1 vs SN2. (D) Heatmaps of 108 significant features shown for one biological replicate of each of the four groups. The color bar represents *z*-scored signal intensities. The green line indicates the time-point of addition of the SN. (E) Time trace of the positive ion at *m*/*z* 98.03173. This feature was significantly different between DC+ SN1 and SN1 as well as significantly different between DC + SN2 vs SN2. It represents an example of a feature with decreased abundance in samples containing DCs.

In study I we investigated whether the method can detect VOCs emitted by DCs and furthermore if the technique discriminates differences in the cellular response to different bacterial SNs. In total, 71 VOCs were significantly different (*p* ≤ 0.05) between SN1 vs SN1 + DC (Table S1) and 59 significantly different (*p* ≤ 0.05) between SN2 vs SN2 + DC (Table S2), showing a log 2FC ≥ 1 compared to their respective baselines (before SN addition). Taken together, 108 unique VOCs were then used to perform t-distributed stochastic neighbor embedding (tSNE) and hierarchical cluster analysis, as illustrated in Figure 2. The variables included in the tSNE show a clear segregation between the four groups and their respective baselines (Figures 2A and S1A). Score 1 over time mainly discriminates between samples containing DCs and samples containing SN only (Figure 2B), whereas score 2 separates SN1 from SN2 (Figure 2C). The heatmaps of the cluster analysis (Figures 2D, S1B, and S1C) interestingly revealed two time trace patterns. On one hand, some features decreased over time in samples containing DCs, as exemplified for *m*/*z* 98.03173 in Figure 2E, compared to samples containing SN only, indicating uptake and/or active metabolism by DCs. On the other hand, a few features increased over time in samples containing DCs, as exemplified for *m*/*z* 182.00907 in Figure S1D compared to SN1 samples, compatible with active production and/or release by DCs. Such behavior may be explained by consumption of certain substrates required for maintenance of cell functions and active cell metabolism when DCs are in an activated vs steady state (e.g., increased glycolysis).^21,22^ Interestingly, besides *m*/*z* 182.00907, we also found two further features with increased abundance in DC + SN1 compared to SN1 over time (Figure S1D). These two features, at *m*/*z* 183.01252 and *m*/*z* 184.00495, were confirmed to be the isotopes of *m*/*z* 182.00907 via isotopic pattern matching (Figure S2). Via LC-MS/MS analysis (Figure S3), the compound was identified as 2-(methylthio) benzothiazole (SCH3-BTH). Benzothiazoles (BTH) represent a class of chemicals widely used in industry, but they are also present, for example, in tap water or indoor dust.^23^ Despite their ubiquitous nature, BTH have also been detected in the exhaled breath of humans, and it has been shown that they are not just artifacts from room air, but also from an endogenous origin.^24^ Interestingly, BTH-based compounds also feature promising antimicrobial activities.^25^ Although the relevance of the higher abundance of SCH3-BTH in the samples with SN1 + DC remains unclear, it suggests an active production of this compound by DCs which may be related to an active antibacterial strategy adopted by the cells.

Intra-coefficient of variation (CV) was assessed during baseline measurements (DCs in medium, *n* = 6 samples with 3 repeated measures each) for compound SCH3-BTH and showed very low intra-experimental variation of ∼7%, which is comparable to a standard Seahorse XF Pro Analysis showing an intraplate CV ≤ 15%.^26^ Therefore, the method shows high technical and good biological reproducibility (e.g., heatmaps Figures 2D, S1B, and S1C). As the experiments were performed with three different biological replicates and on three different days, we could rule out the possibility that our observations resulted from a batch effect. Importantly, cell integrity during real-time measurement is preserved, as shown in Figure S4. Cells after headspace analysis of 6 h showed the same viability (∼95%) when compared to DCs kept in an incubator over the same time period. Another important point to highlight in terms of efficiency is the possibility of measuring multiple conditions (e.g., samples and controls) in one run, which reduces day-to-day variability and overall lead time of experiments.

### Study II: Monitoring ^13^C-Incorporation from Labeled Glucose into VOCs in Real-Time

**Figure 3:**
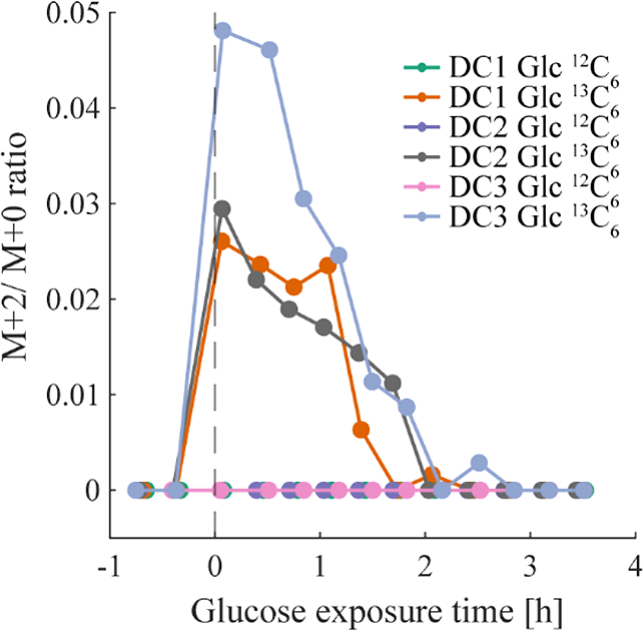
Real-time metabolic ^13^C-incorporation in VOCs emitted by DCs. Real-time ^13^C-incorporation for three DC cell batches exposed to Glc ^13^C_6_ was observed for the feature at *m*/*z* 75.04404 (lactaldehyde) with MF [C_3_H_7_O_2_]^+^, whereas incorporation for DCs exposed to Glc ^12^C_6_ remained at natural abundance. This was consistent for all biological replicates with 5 × 10^6^ cells for DC1 and DC2 and 10^7^ cells for DC3.

After we have shown that the method is able to distinguish between different experimental conditions with high reproducibility, we sought to determine whether the technique is able to detect ^13^C-incorporation into volatile metabolites in real-time over 4 h. Figure 3 illustrates the ^13^C/^12^C ratio time trace of the feature at *m*/*z* 75.04404 obtained for three biological replicates (DC1–3) exposed to Glc ^13^C_6_ or standard glucose (Glc ^12^C_6_), respectively. The feature shows incorporation of two ^13^C with the highest ^13^C/^12^C ratio detected directly after glucose addition, followed by a decreasing ratio close to zero after ∼2 h. As expected, ^13^C/^12^C ratios of samples containing DCs with Glc ^12^C_6_ remained constant at natural abundance level. *m*/*z* 75.04404 was putatively assigned to the compound lactaldehyde which plays a role in pyruvate and carbohydrate metabolism, two core metabolic pathways. However, it must be mentioned that only one feature showed consistent ^13^C-incorporation (according the criteria defined in the data analysis of study II), which may be explained by the fact that DCs initially rely on intrinsic glycogen stocks to sustain metabolic functions and that a certain time-span is required for intracellular metabolites to reach isotopic steady state, especially for nonreplicating cells with lower metabolic activity.^27,28^ Study II shows a high reproducibility among conducted measurements using different biological replicates and demonstrates the feasibility to trace ^13^C-incorporation into volatile metabolites emitted by DCs in real-time. Importantly, this technique can be expanded to beyond ^13^C tracers and potentially accommodate simultaneous tracing of multiple substrates with different labels (e.g., deuterium and ^13^C).

### Study III: ^13^C-Incorporation with and without Long-Time LPS Stimulation

**Figure 4:**
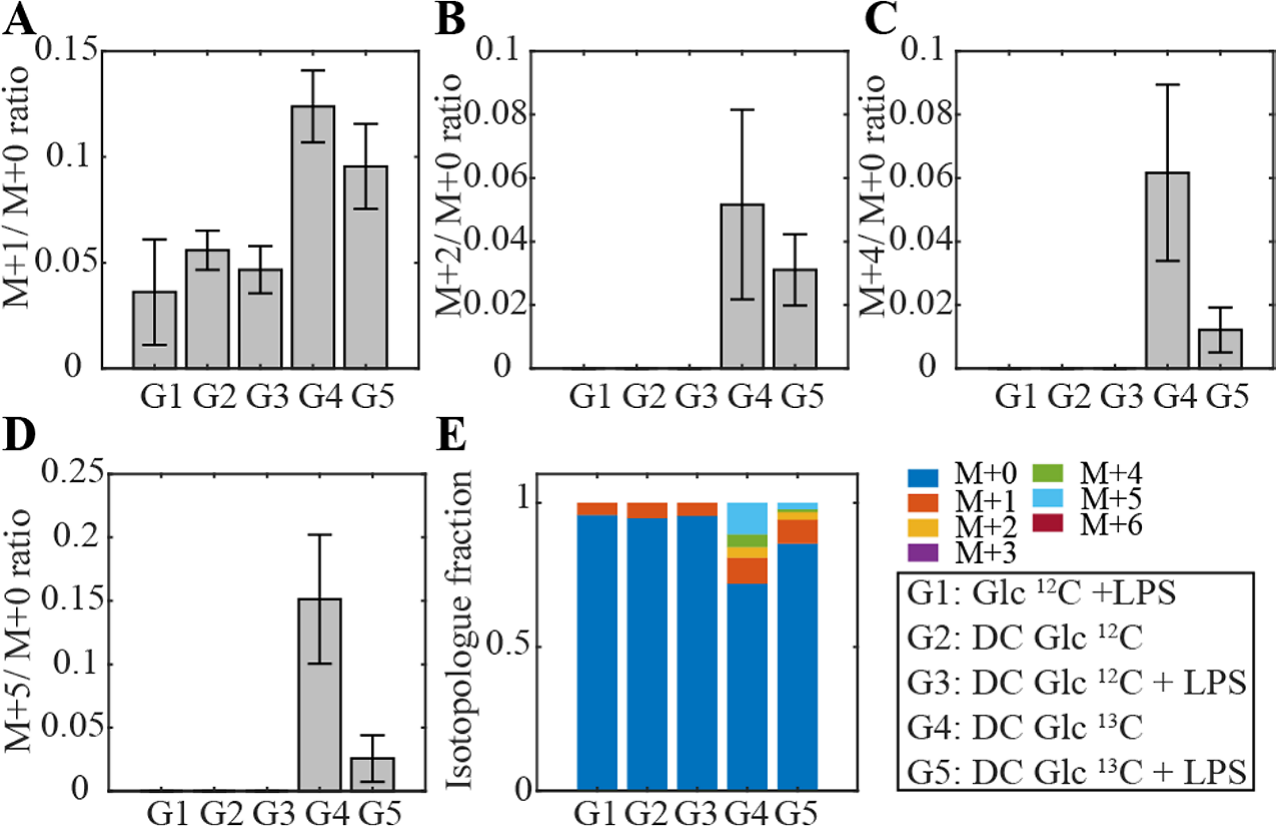
^13^C-incorporation illustrated for the example feature at *m*/*z* 181.07176 with MF [C_6_H_13_O_6_]^−^ detected in negative ionization mode after 24 h stimulation with LPS, shown for all five groups. (A) Incorporation of one, (B) two, (C) four, and (D) five ^13^C was observed in DCs exposed to Glc ^13^C_6_, whereas the incorporation was higher in naïve DCs (G4) than in activated DCs (G5). As expected ^13^C-incorporation in G1, G2, and G3 remained at the natural abundance level. (E) Isotopologue distribution is shown for all groups. Error bars indicate standard deviation (*n* = 4).

Since the given method allows real-time monitoring of ^13^C-incorporation into VOCs, we next assessed whether the technique detects different metabolic states of DCs. It has been shown previously that LPS simulation rewires DC metabolism within 24 h.^22^ Therefore, DCs were exposed to Glc ^13^C_6_ with and without LPS for 24 h. In addition, we also included control groups with DCs exposed to Glc ^12^C_6_ to account for the natural abundance of ^13^C (Figure 4, conditions G1, G2, and G3). We found 22 features (Table S3)/63 isotopologue pairs (Figures 4 and S5) showing significant incorporation (*p* ≤ 0.05) of one up to several ^13^C in G4 or G5 vs G1, G2, and G3. Figure 4 depicts an example of metabolic ^13^C-incorporation for the negative ion at *m*/*z* 181.07176. Incorporation of one, two, four, and five ^13^C was observed (Figure 4A–D). Moreover, the isotopologue distribution (Figure 4E) reflects the metabolic reprogramming of DCs upon LPS-stimulation (G5) compared to immature counterparts (G4). This is especially evident in the M + 4 and M + 5 isotopologue fraction of the two groups for the negative ion at *m*/*z* 181.07176 (Figure 4C,D). As expected, ratios for G1, G2, and G3 remained at a natural abundance level. *m*/*z* 181.07176 was putatively assigned to a sugar (e.g., sorbitol, galacticol) involved in fructose/mannose or galactose metabolism.

**Figure 5:**
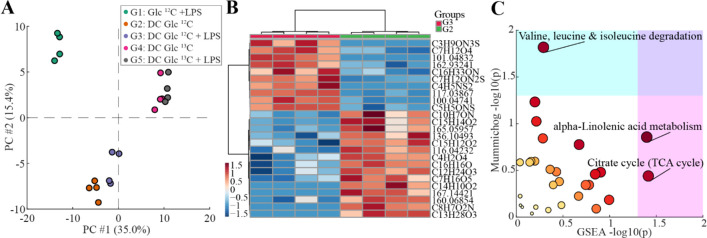
Differences between naïve and activated DCs by LPS stimulation. (A) PCA of significant features (raw *p* ≤ 0.05) that are different between the five groups after ANOVA. (B) Heatmap showing the top 25 different features between G2 and G3. (C) Scatter plot for altered pathways between naïve (G2) and activated DCs (G3). Color and size of the circles correspond to the statistical significance of combined *p*-values from both algorithms.

To get a global overview of the five groups, principal component analysis (PCA) was conducted. Figure S6 shows a PCA considering all features. A clear separation of G1 (without DCs) from the other groups and a slight clustering of the replicates according to group is visible. Analysis of variance (ANOVA) was then conducted and revealed 865 features significantly different (raw *p* ≤ 0.05) between the five groups. Figure 5A depicts a PCA including the significant features and a clear distinction between all five groups is visible. Again, the separation of G1 from the other groups is evident. A separation of G2 and G3 from G4 and G5 is also visible, mainly driven by ^13^C isotopes. As differences in the isotopologue distribution between naïve (G4) and activated (G5) DCs were visible, we then used G2 and G3 (naïve and activated DCs exposed to Glc ^12^C_6_) for PCA visualization (Figure S7), which revealed a clear separation between the two groups. Furthermore, we ran a paired *t* test in order to identify features significantly different between the two groups. We found 9 features which were significantly different (fdr adj. *p* ≤ 0.05) between G2 and G3. We then visualized the top 25 most relevant features in a heatmap (Figure 5B and Table S4). Afterward, we conducted a pathway enrichment analysis to see which pathways may be altered between the two groups. A total of 29 pathways were altered between naïve and activated DCs (Table S5). The top three pathways significantly altered (*p* ≤ 0.05) in either mummichog or GSEA algorithm included: (i) α-linolenic acid metabolism, (ii) valine, leucine, and isoleucine degradation, and (iii) TCA cycle (Figure 5C). Rewiring of the TCA cycle, such as accumulation of TCA intermediate metabolites in activated DCs, has been previously reported.^22^ A further altered pathway was the degradation of essential branched chain amino acids (BCAAs) valine, leucine, and isoleucine. Previous studies indicate that AA play an important role in regulating DC-function.^29^ For example, enhanced uptake of valine, leucine, and isoleucine has been shown in monocyte-derived DCs upon LPS stimulation which fits well with our findings from the enrichment analysis.^30^ Furthermore, depletion of BCAAs from the culture media led to an impaired maturation upon LPS stimulation.^31^ α-Linolenic acid metabolism was another metabolic pathway altered between the two groups. α-Linolenic acid belongs to the group of omega (ω)-3 long-chain polyunsaturated fatty acids (LCPUFAs). Intracellular signaling pathways related to ω-3 LCPUFAs are largely unknown, however, a recent study has shown that ω-3 LCPUFAs, mediated via DCs *in vitro* and *in vivo*, suppressed T-cell proliferation, which suggests that inflammation mediated by T-cells is attenuated.^32^ Taken together, study III exemplifies how this novel method in combination with labeled substrates can be used to gain enhanced insights into VOCs emitted from DCs and underlying metabolic pathways.

Certain limitations with regard to the technical setup need to be considered. This includes the nonautomatized switch of the valves between cell culture flasks (automation would make the data acquisition and measurement procedure more efficient). Moreover, evaporation of the cell culture medium might be observed during substantially long experimental runs, therefore, a sufficient amount of medium, along with constant humidification, must be ensured during the experimental runs. Furthermore, studies I and II were only performed in positive ionization mode, reducing the panel of features that could have been detected. Finally, the identity of the features presented needs to be confirmed, and the interpretation of their potential role in metabolic pathways needs to be further investigated. As the focus of this study was on the technical aspect, this was not within the scope of the current study.

## Conclusion

The proposed mass spectrometric platform allows the monitoring of metabolic trajectories emitted by DCs in real-time. The setup poses an attractive complementary approach to standard metabolomics methods due to its short analysis time, sensitive detection, minimal sample preparation, and high efficiency in measuring multiple probes simultaneously. In combination with labeled substrates, the technique has the potential to provide new insights into metabolic pathways that play a key role in immunological responses triggered by different types of cells.

## Supporting Information Available

Additional details on experiments, materials, methods, data analysis, including supplemental figures and tables (PDF)
