# Supplementary material for: Real-Time Volatile Metabolomics Analysis of Dendritic Cells
Source: Anal Chem. 2023 Jun 13;95(25):9415–21. doi: 10.1021/acs.analchem.3c00516 (PMC10308329; doi:10.1021/acs.analchem.3c00516)
Supplement: Supplementary file 1 — ac3c00516_si_001.pdf [file ac3c00516_si_001.pdf]

Real-time volatile metabolomics analysis of dendritic cells

Kim Arnold[1,2,9], Philippe Dehio[3,9], Jonas Lötscher[3], Kapil Dev Singh[1,2], Diego García-Gómez[4], Christoph Hess[3,5], Pablo Sinues[1,2,10], Maria L. Balmer[3,6,7,8,10]

[1] University Children's Hospital Basel (UKBB), 4056 Basel, Switzerland

[2] Department of Biomedical Engineering, University of Basel, 4123 Allschwil, Switzerland

[3] Department of Biomedicine, Immunobiology, University of Basel and University Hospital of Basel, 4031 Basel, Switzerland

[4] Department of Analytical Chemistry, Nutrition and Food Science, University of Salamanca, 37008 Salamanca, Spain

[5] Department of Medicine, CITIID, Jeffrey Cheah Biomedical Centre, University of Cambridge, Cambridge CB2 0AW, UK

[6] Department of Biomedical Research (DBMR), University of Bern, 3008 Bern, Switzerland

[7] University Clinic for Diabetes, Endocrinology, Clinical Nutrition and Metabolism, Inselspital, 3010 Bern, Switzerland

[8] Diabetes Center Bern (DCB), 3010 Bern, Switzerland

[9] These authors contributed equally to this paper

[10] These senior authors contributed equally to this paper

Table of content

SESI-HRMS data analysis S3

LC-MS/MS analysis for compound identification of SCH_3-BTH S8

Flow cytometric viability analysis S8

Figure S1 .... S10

Figure S2 .... S11

Figure S3 .... S12

Figure S4 .... S13

Figure S5 .... S14

Figure S6 .... S16

Figure S7 .... S17

Table S1 .... S18

Table S2 .... S20

Table S3 .... S22

Table S4 .... S23

Table S5 .... S24

References S25

SESI-HRMS data analysis

Data acquisition was completed over a time-span of two years (2020 – 2022). The data preprocessing sections are different among experiments because the data preprocessing pipeline was continuously improved.

Data preprocessing – Study I and Study II

Data preprocessing of studies I and II was conducted according to our patented data processing pipeline (European patent No. 20186274.5 and 21185400.5) using MATLAB (version 2021b, Mathworks Inc., USA). To summarize, RAW files were accessed via in-house C# console apps based on Thermo Fisher Scientific's RawFileReader (version 5.0.0.38). To generate the feature list for each experiment, the MATLAB function *ksdensity* was used for binning the centroid peak list from each scan of all files. XIC were extracted for all features and TIC from each measurement file. For subsequent analysis, only peaks with a signal intensity above 10e3 a.u. in Thermo's signal intensity scale were further considered when isotopic labeling experiments were performed (study II), or peaks with a signal intensity above 10e4 a.u. for experiments performed in study I. In case satellite artefact peaks were present after applying the defined peak cutoff, those *m/z* regions were excluded from further analysis (i.e., study I: *m/z* 50-54, *m/z* 59-60, *m/z* 97-98 and *m/z* 118-119; study II: *m/z* 50-61, *m/z* 72, *m/z* 96-97). Time windows when the samples were pervaded with the gas mixture were determined via a z-scored TIC cutoff. After this, the area under the curves (AUC) during the defined time windows when gas supply through the samples was provided were numerically integrated. As a last step, normalization of AUCs by the time window was performed to get the time-normalized area under the curve (nAUC). Molecular formulae (MF) were assigned based on the "seven golden rules"[1] considering the elements C, H, O, N and S and the adduct $[M+H]^+$. MF-assignment according to the "seven golden rules"[1] is based on an algorithm derived from seven heuristic rules which

ensures correct elemental compositions and assigns the most likely and chemically correct MF to a mass spectral feature of interest.

Data preprocessing – Study III

MATLAB (version R2022a, MathWorks, USA) was used for data preprocessing. Profile mass spectra and raw centroids (intensity cut-off = 50^2 a.u.) were retrieved by using in-house C# console apps based on Thermo Fisher Scientific's RawFileReader (version 5.0.0.38). Afterwards, reference peaks with formulae fulfilling the "seven golden rules"[1] and common contaminants[2] present in at least 50% of the samples were used to recalibrate centroid and profile mass spectra applying an initial tolerance of 5 ppm. Subsequently, an in-house shape-preserving piecewise cubic interpolation algorithm was deployed to estimate the experimental error across the whole *m/z*-range. Observed outliers assessed for the reference peaks by moving median algorithm, were excluded when interpolation was performed. This was followed by shifting of the centroids and profile peaks according to the obtained mass error. This process was applied three times for the whole *m/z*-range of all mass spectra to make sure that the mass error falls below 0.5 ppm. To bin the histograms of the recalibrated centroid peak list, kernel density function was applied. To ensure Gaussian probability density functions of +/- 1 ppm at full width at half maximum, an iteration on the bandwidth controlling the smoothness of the probability density curve was performed. Lastly, to create the final data matrix, centroids present in the previously mentioned window were used. Only $m/z \geq 100$ were used for subsequent analysis to exclude satellite artefact peaks. MF were assigned based on the "seven golden rules"[1] considering the elements C, H, O, N and S and the adduct $[M+H]^+$ in positive mode and $[M-H]^-$ in negative mode.

Data postprocessing – Study I

As a result, from preprocessing, a data matrix of total 216 files (18 files/time points per sample) x 4104 mass spectral features in positive mode was obtained. Time traces for each feature were then computed. Next, the time-normalized AUC of each time trace was calculated for each of the three biological replicates before and after SN addition. To identify differences between the four sample groups after SN addition, a one-way analysis of variance (ANOVA) with AUCs was performed, followed by a posthoc multiple comparison using Tukey's significant difference procedure. For further statistical evaluation only significant features ($p \leq 0.05$) between the sample groups of SN1 vs. SN1 + DC and SN2+DC vs. SN2 were further considered. As a second criteria, only features with a log2 fold change (log2FC) ≥1 in mean area under the curve (mAUC) after SN vs. before SN addition were considered as relevant. The data matrix of the remaining features including only the 15 time points after SN addition was then 5th-root transformed and used to perform t-distributed stochastic neighbor embedding (tSNE) (i.e., Figure 2A and Figure S1A). In addition, the whole data matrix was auto-scaled (z-score) and subjected to hierarchical cluster analysis (Ward method; Euclidean distance) and visualized as heat maps (i.e., Figure 2D, Figure S1B and Figure S1C).

Data postprocessing – Study II

As a result of preprocessing, a data matrix of total 78 files (13 files/time points per sample) x 4384 mass spectral features in positive mode was obtained. Afterwards, features were only considered for further analysis if their signal intensity was greater than zero in at least five out of 13 data time points measured for the samples containing Glc $^{13}C_6$. $^{13}C/^{12}C$ isotopologue pairs were identified by using a mass difference $\Delta = n \times 1.0034u$ ($\pm$ 0.0005u), where n =1, 2, . . . 10 and u = unified atomic mass unit. After this the $^{13}C/^{12}C$ ratios were computed for all samples at all time points. Feature ratio time traces containing not a number (NaN) values and higher

ratios than expected for natural abundance (natural abundance $^{13}C/^{12}C$ ratio = 0.01109) in samples containing Glc $^{12}C_6$ were dropped (i.e., ratios > 0.1 as some tolerance due to measurement variability was given). Furthermore, only those features for which the average signal intensity ratios of the samples containing Glc $^{13}C_6$ were at least twice as high compared to the average signal intensity ratios of the samples containing Glc $^{12}C_6$ were further considered. This ensured that those features showing an increasing ratio trend and therefore an incorporation of ^{13}C were captured. Features where the suggested number of incorporated ^{13}C was higher than the proposed MF were not further considered. Only those features which showed consistent ^{13}C-incorporation among all three biological replicates were finally used for visualizing the $^{13}C/^{12}C$ ratio time traces (i.e., Figure 3).

Data postprocessing – Study III

A data matrix of total 20 files (4 biological replicates/group) x 2759 mass spectral features in positive mode and x 938 features in negative mode was obtained. $^{13}C/^{12}C$ isotopologue pairs were identified by using a mass difference $\Delta = n \times 1.0034u$ ($\pm$ 0.0005u), where n =1, 2, . . . 10 and u = unified atomic mass unit. Next, the signal intensity ratios $^{13}C/^{12}C$ were computed for all samples in positive and negative mode. Calculated ratios resulting in NaN values were excluded. ANOVA was then performed for positive and negative ratios of the five groups separately, followed by a post hoc multiple comparison using Tukey's significant difference procedure. Only features fulfilling the following criteria were further considered relevant in negative or positive mode: I) G1, G2 and G3 must be significant ($p \leq 0.05$) vs. G4 and G5 or II) G1, G2 and G3 must be significant ($p \leq 0.05$) vs. G4 or III) G1, G2 and G3 must be significant ($p \leq 0.05$) vs. G5. In addition, only features showing a positive difference in the estimated group means of G4 and G5 vs. G1, G2 and G3 were further considered. Furthermore, if for the significant features, the $^{13}C/^{12}C$ ratio in G1, G2 and G3 was higher than expected natural abundance (see data postprocessing study II), the features were not further considered. Features

where the suggested number of incorporated ^{13}C was higher than the proposed MF were also not considered for the final ratio plots (Figure 4, Figure S5 and Table S3).

To perform PCA, original positive and negative matrices were merged. For PCA visualization including all features, data were first log10-transformed and afterwards auto-scaled (Figure S6). For PCA visualization including only significant features, a one-way ANOVA test was performed on the log10-transformed data matrix. The data matrix containing the significant features (raw $p \leq 0.05$) was then auto-scaled and used to perform PCA (Figure 5A).

Positive and negative data matrices of G2 and G3 were used for statistical analysis and feature selection via MetaboAnalystR (version 5.0; https://www.metaboanalyst.ca). Data were log10 transformed and pareto-scaled (mean-centered and divided by the square root of the standard deviation of each variable). PCA was then used to first visualize the data (Figure S7). Statistical analysis was performed using a paired t-test. The top 25 features (Table S4) were then used for cluster analysis and illustrated as a heatmap (Figure 5B). Furthermore, raw p-values after paired t-test and log2FC were used to perform metabolic pathway enrichment analysis (Figure 5C, Table S5) via the "MS Peaks to Pathways" module of MetaboAnalstR (version 5.0) using the following settings: mass accuracy = 5 ppm, instrument mode = mixed, method version = v1, organism library = mmu_kegg, library version = current, mummichog p-value cutoff = 0.05, minimum number of metabolites needed to consider the pathway = 3, permutation number = 100. The adducts M+H [1+] and M(C^{13}) + H [1+] were used for compound assignments in positive mode and M-H [1-] and M(C^{13})-H [1-] in negative mode.

Putative compound assignment of features presented in Figure 3 and Figure 4

Putative compound assignment was done in MetaboAnalyst R (version 5.0) using mmu_kegg organism library considering only protonated and deprotonated species within 5 ppm.

LC-MS/MS analysis for compound identification of SCH_3-BTH

At the end of the volatilome measurements performed in study I with bacterial SN, the content of one cell culture flask containing DCs with SN1 was transferred to a Falcon tube and centrifuged for 5 min at 1500 rounds per min (rpm). After centrifugation, 3 mL of the supernatant was transferred to an Eppendorf tube and stored at -80°C until LC-MS analysis. Standards and samples were analyzed by means of a Vanquish UHPLC system equipped with a Q Exactive Focus Orbitrap spectrometry detector (Thermo Fisher Scientific, Waltham, MA, USA). SCH_3-BTH was separated in a CORTECS C18 column (2.7 µm, 2.1 x 150mm) from Waters Corporation (Milford, MA, USA) with a mobile phase formed by a mixture of (A) water with 0.1% formic acid and (B) acetonitrile with 0.1% formic acid flowing at 0.4 mL/min with a gradient from 20% to 25% B in 4 minutes, followed by an increase to 70% B in 1 minute, withhold for 4 min and a re-equilibration time of 1 min to initial conditions for a total runtime of 10 min. Injection was set to 50 µL and the column temperature to 25ºC. Electrospray ionization was carried out at 4kV. Nitrogen was used as desolvation gas with sheath, auxiliary and sweep flow rates of 20, 10 and 2 (275ºC), respectively. Two parallel experiments were recorded: a full-mass scan from 50 to 750 u and a product reaction monitoring from 50 to 205 u with a collision energy (HCD) of 37, a fixed resolution of 35,000 and an isolation window of 0.4 u for the *m/z* values 182.00927 (protonated SCH3-BTH) and 184.00512 (^{34}S isotope of protonated SCH3-BTH).

Flow cytometric viability analysis

For the assessment of cell viability, the DCs were removed with a cell scraper from flasks that were connected to the SESI-HRMS platform or kept in a cell culture incubator. The cells were then washed once in PBS buffer and stained with the zombie aqua dye (Biolegend; catalogue #423101) for 15 min at room temperature and washed in PBS + 1% BSA, according to the

manufacturer’s instructions. The stained cells were afterwards acquired with a CytoFLEX flow cytometer (Beckman Coulter).

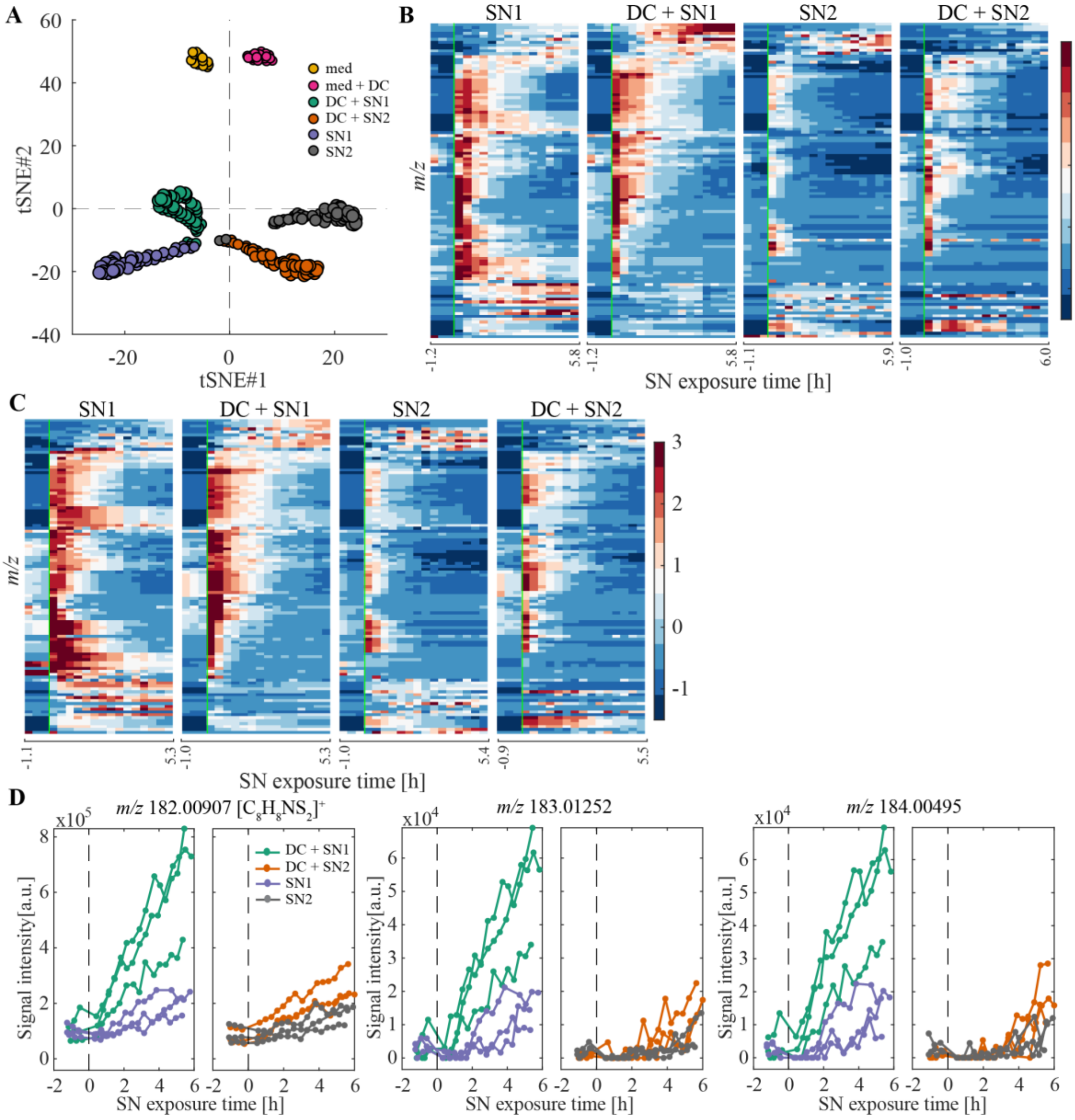

Figure S1. Distinguishing experimental conditions with high technical and biological reproducibility. (A) tSNE plot of the 108 significant features, also showing samples before SN addition (med = medium; med +DC = medium with DCs). (B) Heatmaps of 108 significant features shown for the second biological replicate of each of the four groups. (C) Heatmaps of 108 significant features shown for the third biological replicate of each of the four groups. (D) Example feature *m/z* 182.00907 showing an increased abundance over time in DC samples stimulated with SN1. Feature time traces at *m/z* 183.01252 and *m/z* 184.00495 represent the ^{13}C isotopes of *m/z* 182.00907.

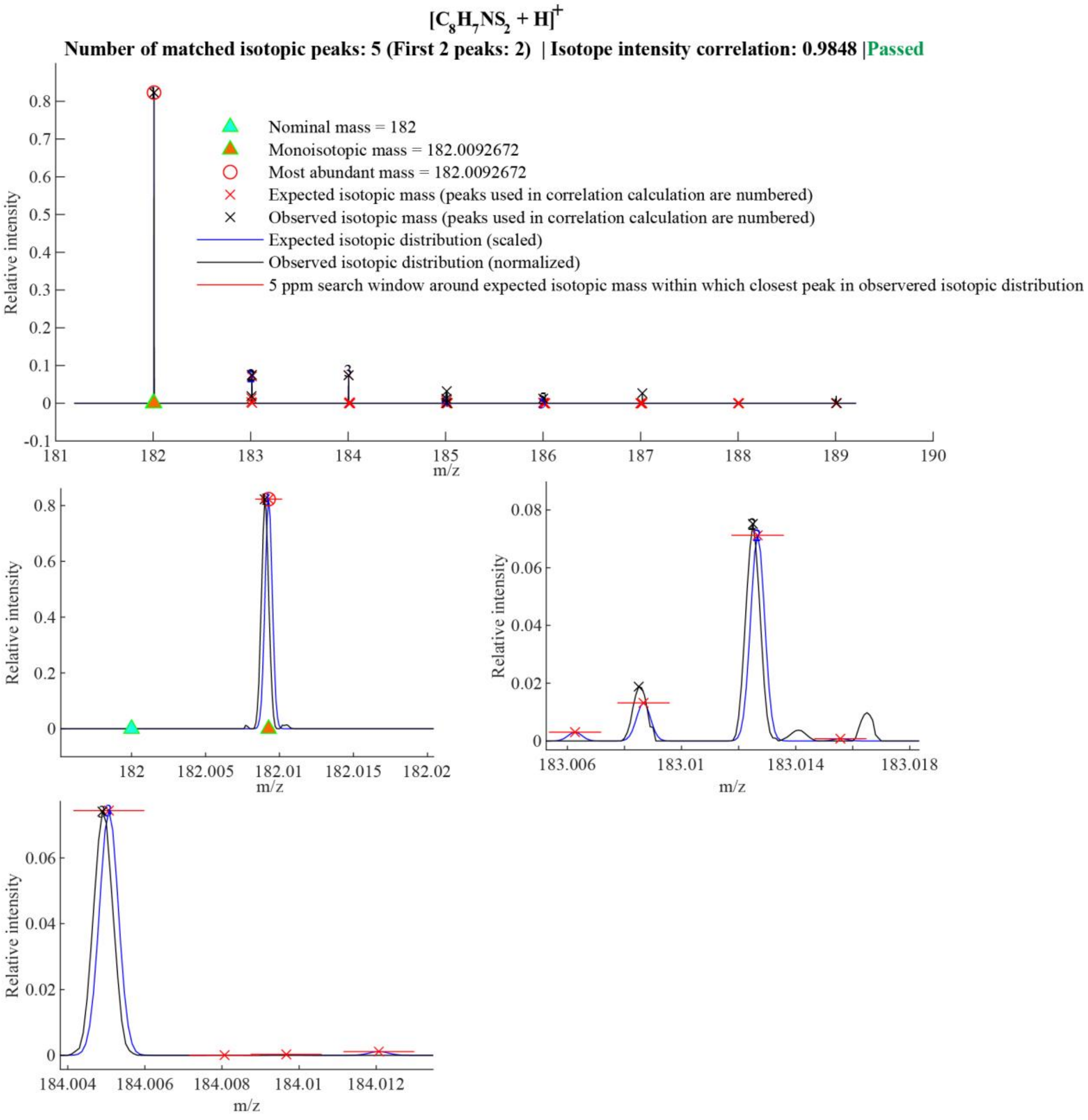

Figure S2. Isotopic pattern matching SCH_3-BTH. A total of five isotopic peaks were matched with *m/z* 182.0092 and MF $[C_8H_8NS_2]^+$. Isotope correlation intensity was calculated according to the first two isotopes of *m/z* 182.00907 (i.e., *m/z* 183.01252 and *m/z* 184.00495). Zoomed-in mass spectra of *m/z* 182.00907, *m/z* 183.01252 and *m/z* 184.00495 show the nice overlap between expected and observed isotopic distribution.

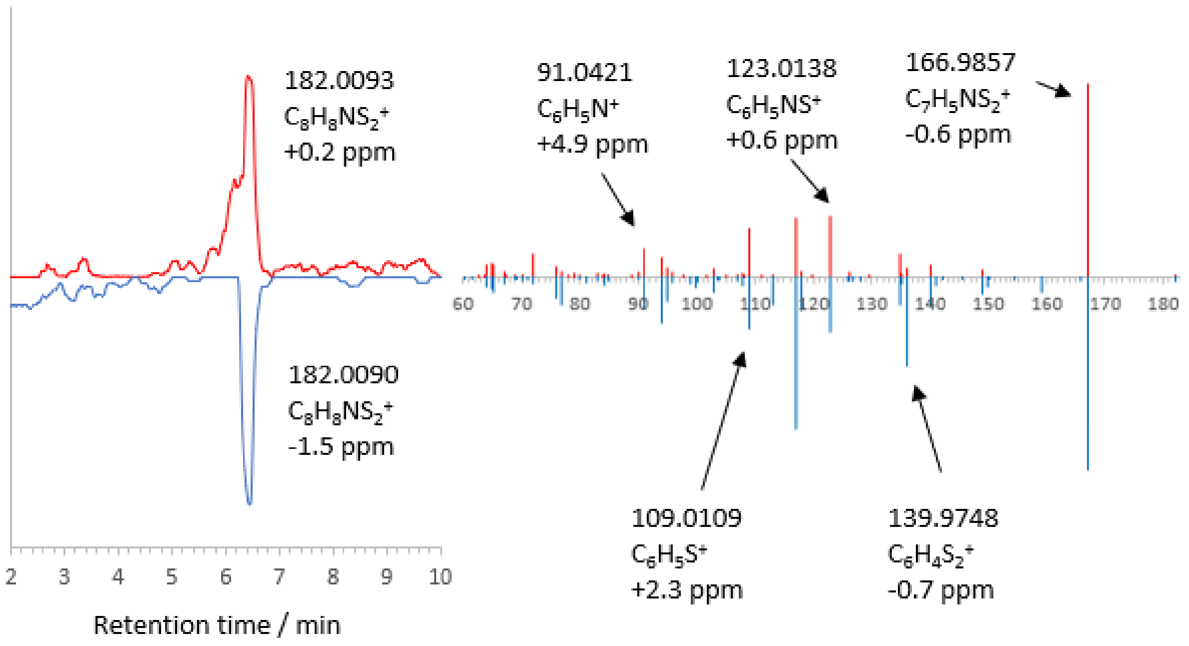

Figure S3. LC-MS/MS confirmation of SCH_3-BTH. Matching retention time along with the matching MS/MS spectra is shown for one DC + SN1 sample after the volatilome analysis (red) and for a standard (blue) at a concentration of 400 ng/L.

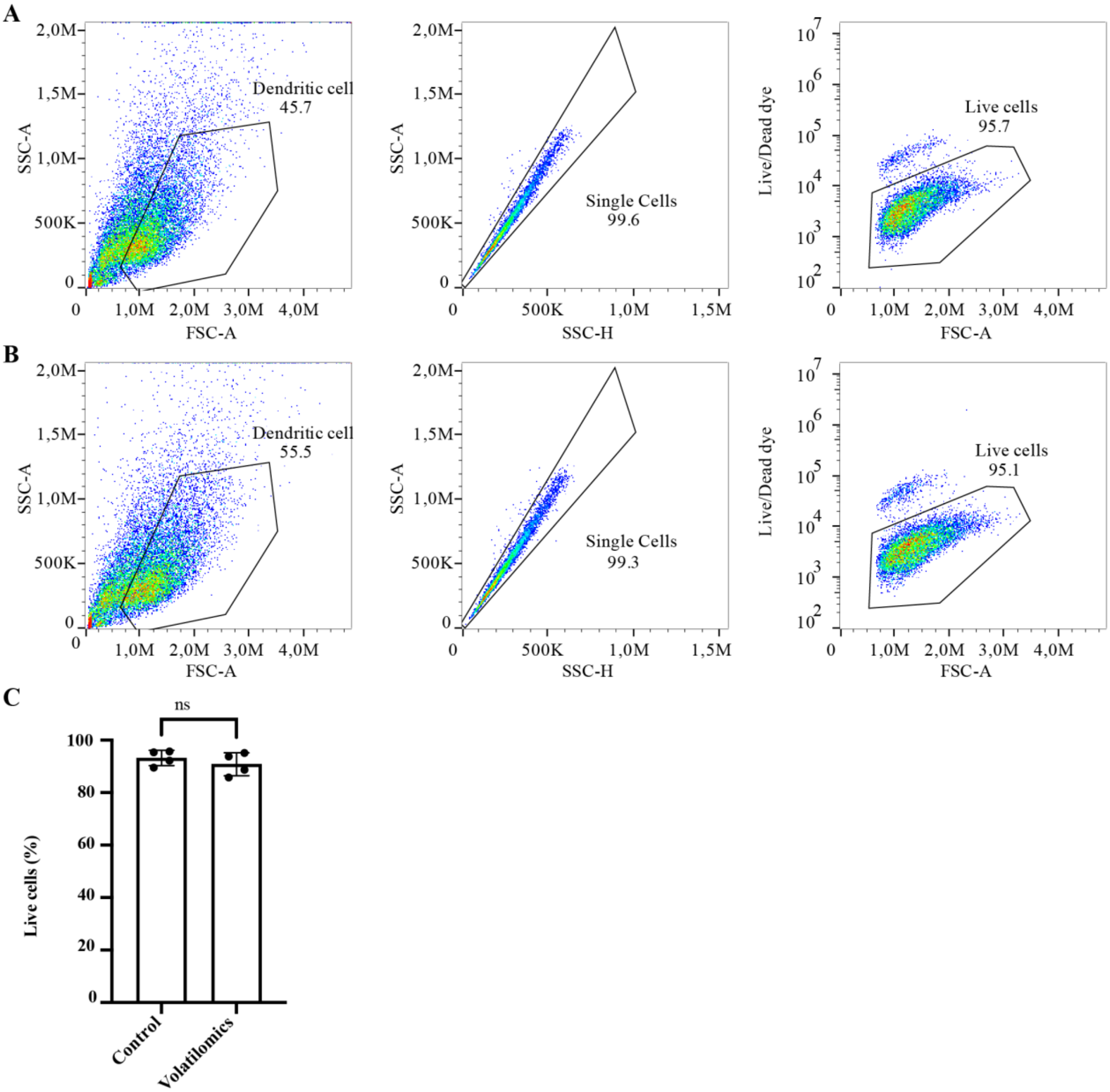

Figure S4. Flow cytometric viability analysis. (A) Results from a cell culture flask kept for 6 h in an incubator at 37 °C and 5% CO^2. (B) Results from a cell culture flask after 6 h real-time headspace analysis using SESI-HRMS. (C) Quantification of living cells (frequency of Live/Dead dye negative cells) indicating no significant difference between the cell culture kept in the incubator vs. cell culture from mass spectrometric analysis which confirms sample integrity during DC headspace analysis.

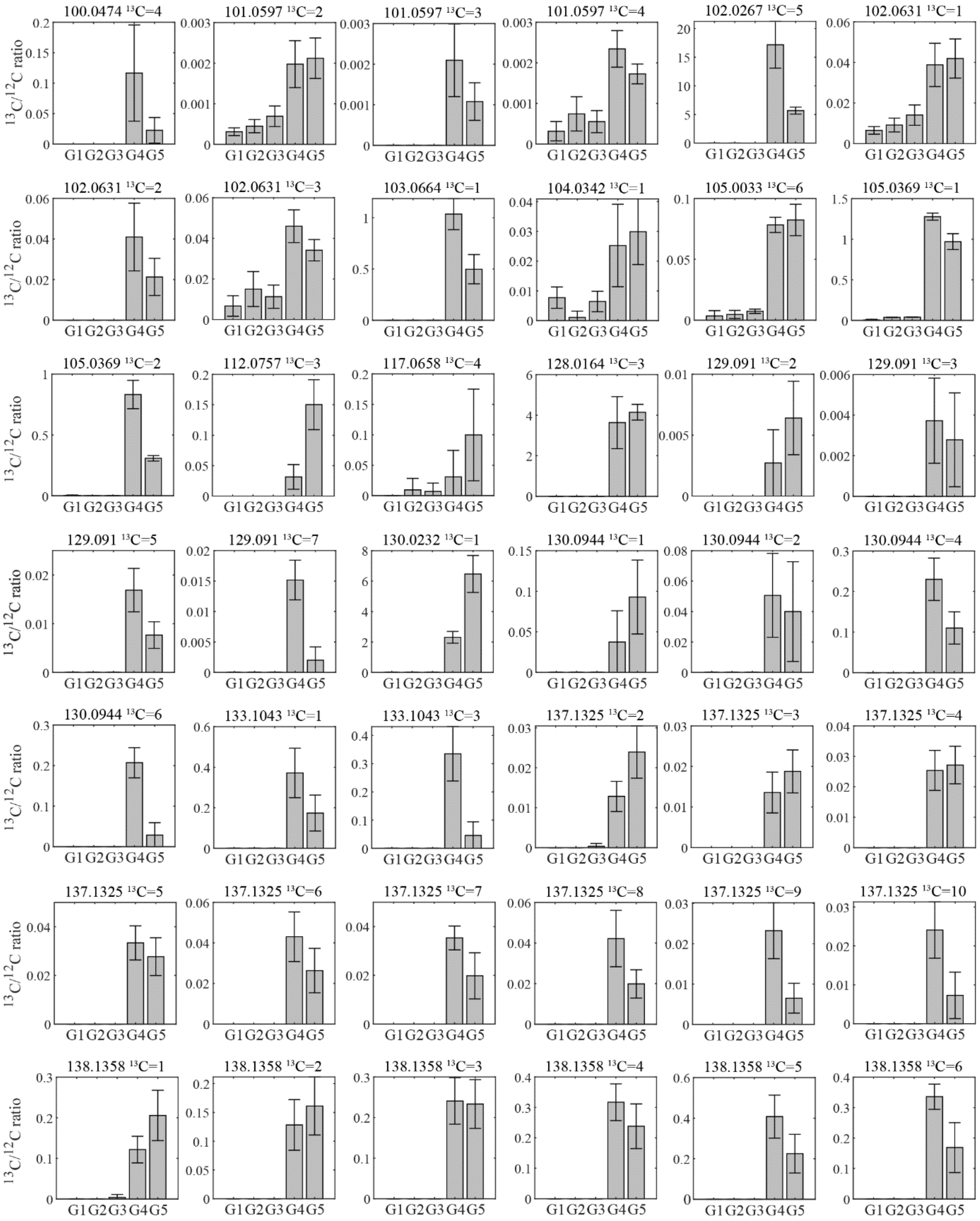
100.0474 13C=4
101.0597 13C=2
101.0597 13C=3
101.0597 13C=4
102.0267 13C=5
102.0631 13C=1
102.0631 13C=2
102.0631 13C=3
103.0664 13C=1
104.0342 13C=1
105.0033 13C=6
105.0369 13C=1
105.0369 13C=2
112.0757 13C=3
117.0658 13C=4
128.0164 13C=3
129.091 13C=2
129.091 13C=3
129.091 13C=5
129.091 13C=7
130.0232 13C=1
130.0944 13C=1
130.0944 13C=2
130.0944 13C=4
130.0944 13C=6
133.1043 13C=1
133.1043 13C=3
137.1325 13C=2
137.1325 13C=3
137.1325 13C=4
137.1325 13C=5
137.1325 13C=6
137.1325 13C=7
137.1325 13C=8
137.1325 13C=9
137.1325 13C=10
138.1358 13C=1
138.1358 13C=2
138.1358 13C=3
138.1358 13C=4
138.1358 13C=5
138.1358 13C=6
13C/12C ratio
G1 G2 G3 G4 G5

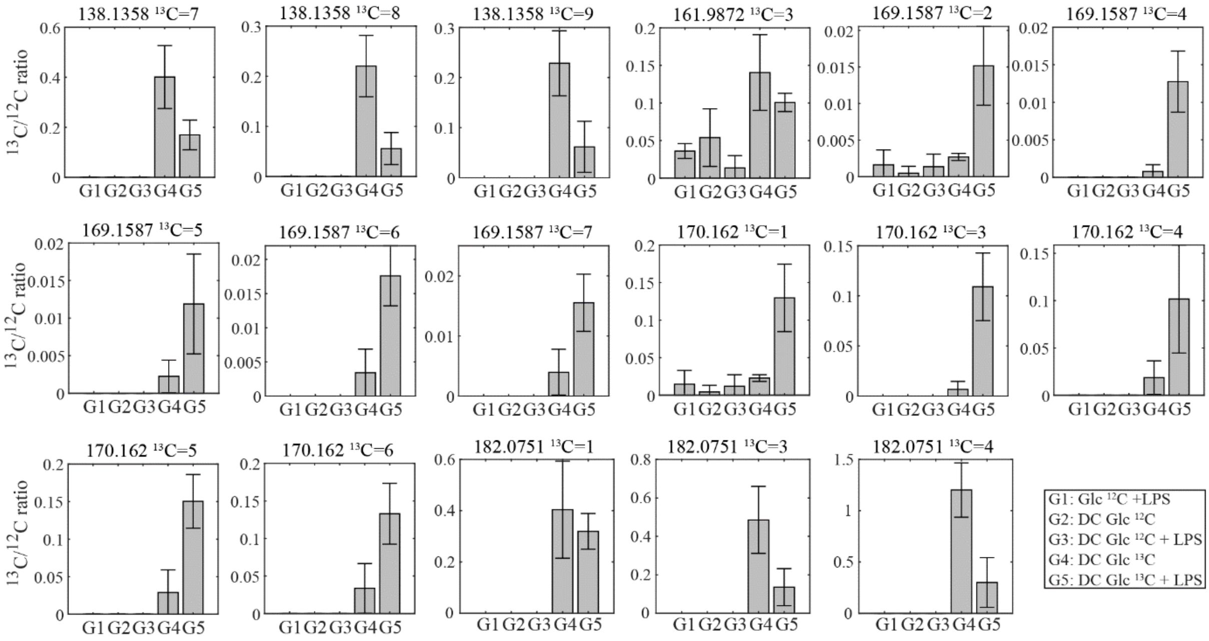

Figure S5. ^{13}C-incorporation illustrated for the remaining significant isotopologues. The title of each plot indicates the *m/z* of the features showing ^{13}C-incorporation as well as the number of incorporated ^{13}C. Error bars indicate standard deviation of the mean (*n*=4).

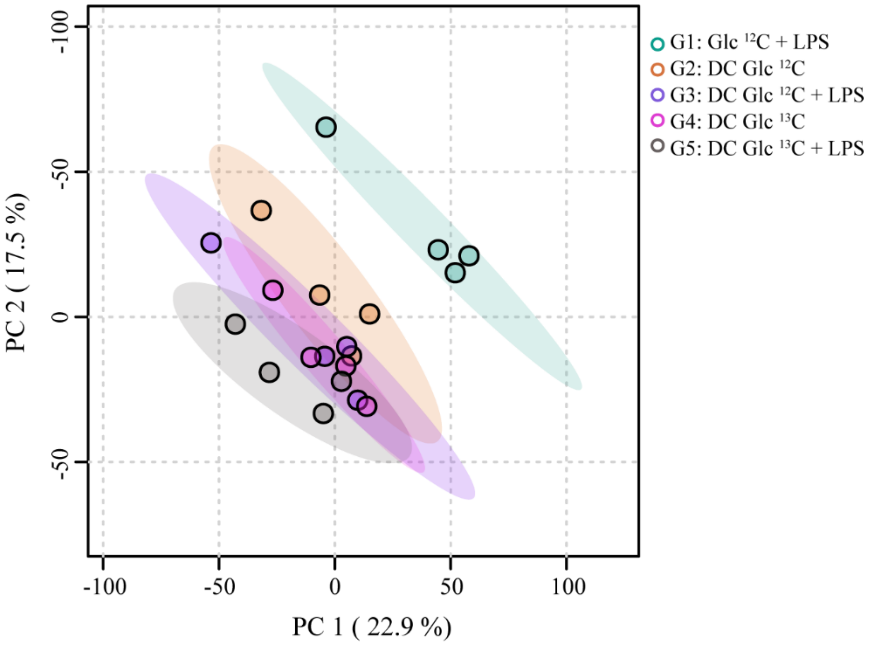

Figure S6. PCA plot including all features depicting inherent differences between the five groups. A total of ~ 40% of variance is explained by the first two PC scores.

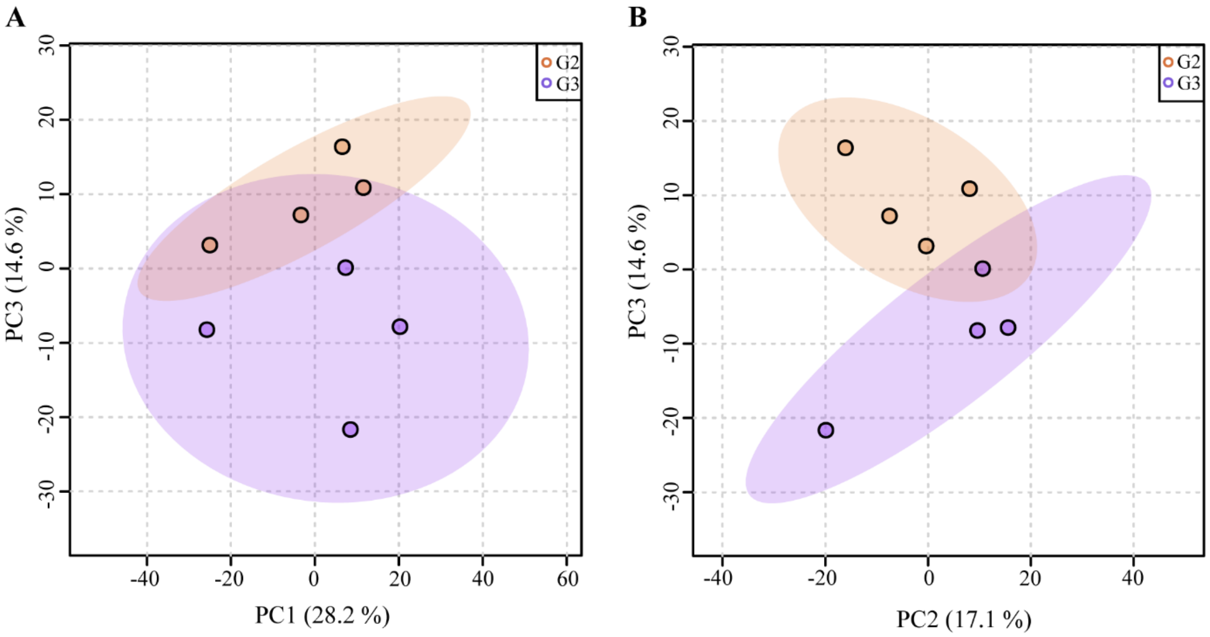

Figure S7. PCA plot depicting differences between naïve (G2) and activated DCs (G3). (A) PCA of score 1 vs. score 3. (B) PCA of score 2 vs. score 3. A total variance of ~60% is explained by the first three scores.

Table S1. 71 significant features different between SN1 + DC and SN1.

m/z[a]	Molecular Formula (M)[b]	Adduct	Mass error (ppm)	p-value
109.05337	NA	$[M+H]^+$	NA	8.12E-11
99.03503	NA	$[M+H]^+$	NA	7.61E-10
183.08371	$C_{10}H_{14}OS$	$[M+H]^+$	-0.549	1.26E-07
109.05582	NA	$[M+H]^+$	NA	2.67E-07
114.00891	NA	$[M+H]^+$	NA	6.87E-06
113.00552	C_5H_4OS	$[M+H]^+$	-0.356	7.60E-06
115.00135	NA	$[M+H]^+$	NA	7.80E-06
107.04913	C_7H_6O	$[M+H]^+$	-0.153	1.48E-05
85.01060	C_4H_4S	$[M+H]^+$	-0.599	1.60E-05
108.05246	NA	$[M+H]^+$	NA	1.74E-05
99.03261	NA	$[M+H]^+$	NA	3.31E-04
98.03173	NA	$[M+H]^+$	NA	5.02E-04
115.00414	NA	$[M+H]^+$	NA	1.21E-03
122.07101	NA	$[M+H]^+$	NA	1.87E-03
115.99658	NA	$[M+H]^+$	NA	1.88E-03
128.05284	C_6H_9NS	$[M+H]^+$	-0.089	2.05E-03
103.05419	C_8H_6	$[M+H]^+$	-0.329	2.40E-03
99.02625	C_5H_6S	$[M+H]^+$	-0.493	2.41E-03
69.05279	NA	$[M+H]^+$	NA	2.46E-03
88.08666	NA	$[M+H]^+$	NA	2.48E-03
137.08711	NA	$[M+H]^+$	NA	3.59E-03
58.04128	NA	$[M+H]^+$	NA	3.95E-03
94.07320	NA	$[M+H]^+$	NA	4.30E-03
183.01252	NA	$[M+H]^+$	NA	4.59E-03
182.00908	$C_8H_7NS_2$	$[M+H]^+$	-1.044	4.74E-03
152.15140	NA	$[M+H]^+$	NA	4.76E-03
194.12561	NA	$[M+H]^+$	NA	4.95E-03
93.06986	C_7H_8	$[M+H]^+$	-0.191	5.09E-03
108.08881	NA	$[M+H]^+$	NA	5.16E-03
111.04404	$C_6H_6O_2$	$[M+H]^+$	-0.116	5.24E-03
114.00082	C_4H_3ONS	$[M+H]^+$	0.063	5.32E-03
184.00495	NA	$[M+H]^+$	NA	5.33E-03
112.04738	NA	$[M+H]^+$	NA	5.62E-03
180.15748	NA	$[M+H]^+$	NA	5.82E-03
87.00290	NA	$[M+H]^+$	NA	7.68E-03
61.01061	C_2H_4S	$[M+H]^+$	-0.605	1.04E-02
113.04190	C_6H_8S	$[M+H]^+$	-0.413	1.05E-02
61.05580	NA	$[M+H]^+$	NA	1.44E-02
127.02122	C_6H_6OS	$[M+H]^+$	0.088	1.52E-02
123.06901	NA	$[M+H]^+$	NA	1.66E-02
60.05560	NA	$[M+H]^+$	NA	1.68E-02
60.05539	NA	$[M+H]^+$	NA	1.71E-02
171.09145	$C_{11}H_{10}N_2$	$[M+H]^+$	-1.297	2.06E-02
95.04911	C_6H_6O	$[M+H]^+$	-0.321	2.25E-02
102.01732	NA	$[M+H]^+$	NA	2.33E-02
152.07863	NA	$[M+H]^+$	NA	2.39E-02
61.05335	NA	$[M+H]^+$	NA	2.41E-02
128.02456	C_7HN_3	$[M+H]^+$	1.877	2.46E-02
107.08549	C_8H_{10}	$[M+H]^+$	-0.345	2.46E-02
60.05334	NA	$[M+H]^+$	NA	2.56E-02
87.08043	$C_5H_{10}O$	$[M+H]^+$	-0.142	2.60E-02
58.99089	NA	$[M+H]^+$	NA	2.67E-02
105.03686	C_4H_8OS	$[M+H]^+$	-0.009	2.67E-02
126.03718	C_6H_7NS	$[M+H]^+$	-0.106	2.69E-02
126.13583	NA	$[M+H]^+$	NA	2.77E-02

m/z[a]	Molecular Formula (M)[b]	Adduct	Mass error (ppm)	p-value
185.10720	$C_{12}H_{12}N_2$	$[M+H]^+$	-0.655	2.82E-02
123.07147	NA	$[M+H]^+$	NA	2.84E-02
88.08375	NA	$[M+H]^+$	NA	2.89E-02
179.15410	$C_{11}H_{18}N_2$	$[M+H]^+$	-0.958	2.90E-02
122.06812	NA	$[M+H]^+$	NA	3.01E-02
136.08670	$C_7H_9N_3$	$[M+H]^+$	-1.648	3.04E-02
129.11843	NA	$[M+H]^+$	NA	3.04E-02
60.05249	NA	$[M+H]^+$	NA	3.08E-02
176.05268	$C_{10}H_9NS$	$[M+H]^+$	-0.944	3.20E-02
101.02488	NA	$[M+H]^+$	NA	3.24E-02
129.01697	NA	$[M+H]^+$	NA	3.42E-02
95.01269	$C_5H_2O_2$	$[M+H]^+$	-0.700	3.95E-02
89.08708	NA	$[M+H]^+$	NA	4.07E-02
153.16369	$C_{11}H_{20}$	$[M+H]^+$	-0.543	4.25E-02
121.06481	C_8H_8O	$[M+H]^+$	0.173	4.61E-02
204.16752	NA	$[M+H]^+$	NA	4.84E-02

[a] Exact mass (*m/z*) of the ion.

[b] Formula based on accurate mass (no isotopic pattern matching performed).

NA = not available.

Table S2. 59 significant features different between SN2 + DC and SN2.

m/z [a]	Molecular Formula (M)[b]	Adduct	Mass error (ppm)	p-value
103.02097	NA	$[M+H]^+$	NA	2.44E-04
87.00290	NA	$[M+H]^+$	NA	4.63E-04
87.00525	NA	$[M+H]^+$	NA	9.48E-04
101.02488	NA	$[M+H]^+$	NA	1.21E-03
86.00584	C_3H_3NS	$[M+H]^+$	-0.615	1.32E-03
100.02148	C_4H_5NS	$[M+H]^+$	-0.720	1.43E-03
88.00164	NA	$[M+H]^+$	NA	1.63E-03
102.01732	NA	$[M+H]^+$	NA	2.06E-03
87.00921	NA	$[M+H]^+$	NA	2.07E-03
139.11170	$C_9H_{14}O$	$[M+H]^+$	-0.313	2.11E-03
140.11502	NA	$[M+H]^+$	NA	2.11E-03
217.14351	$C_{11}H_{20}O_4$	$[M+H]^+$	0.328	2.16E-03
58.04128	NA	$[M+H]^+$	NA	2.58E-03
183.08371	$C_{10}H_{14}OS$	$[M+H]^+$	-0.549	5.12E-03
60.05560	NA	$[M+H]^+$	NA	5.13E-03
60.05539	NA	$[M+H]^+$	NA	5.31E-03
60.05334	NA	$[M+H]^+$	NA	7.55E-03
61.05335	NA	$[M+H]^+$	NA	8.22E-03
114.00082	C_4H_3ONS	$[M+H]^+$	0.063	8.49E-03
60.05249	NA	$[M+H]^+$	NA	8.74E-03
68.04945	C_4H_5N	$[M+H]^+$	-0.322	1.08E-02
225.14857	$C_{13}H_{20}O_3$	$[M+H]^+$	0.239	1.10E-02
61.05580	NA	$[M+H]^+$	NA	1.11E-02
318.24284	$C_{20}H_{31}O_2N$	$[M+H]^+$	0.278	1.18E-02
141.11836	NA	$[M+H]^+$	NA	1.37E-02
126.03718	C_6H_7NS	$[M+H]^+$	-0.106	1.47E-02
80.03678	NA	$[M+H]^+$	NA	1.55E-02
193.12227	$C_{12}H_{16}O_2$	$[M+H]^+$	-0.197	1.61E-02
154.06840	$C_8H_{11}NS$	$[M+H]^+$	-0.610	1.70E-02
171.09145	$C_{11}H_{10}N_2$	$[M+H]^+$	-1.297	1.70E-02
119.03724	NA	$[M+H]^+$	NA	1.83E-02
168.08395	$C_9H_{13}NS$	$[M+H]^+$	-1.175	1.96E-02
114.03720	C_5H_7NS	$[M+H]^+$	0.068	2.03E-02
98.03173	NA	$[M+H]^+$	NA	2.25E-02
199.12291	$C_{13}H_{14}N_2$	$[M+H]^+$	-0.351	2.31E-02
195.13792	$C_{12}H_{18}O_2$	$[M+H]^+$	-0.184	2.36E-02
151.14806	$C_{11}H_{18}$	$[M+H]^+$	-0.424	2.54E-02
185.10720	$C_{12}H_{12}N_2$	$[M+H]^+$	-0.655	2.64E-02
103.05419	C_8H_6	$[M+H]^+$	-0.329	2.67E-02
109.35339	NA	$[M+H]^+$	NA	2.67E-02
99.03261	NA	$[M+H]^+$	NA	2.74E-02
225.18496	$C_{14}H_{24}O_2$	$[M+H]^+$	0.242	2.88E-02
150.09939	NA	$[M+H]^+$	NA	2.93E-02
189.18479	$C_{11}H_{24}O_2$	$[M+H]^+$	-0.642	2.94E-02
115.99658	NA	$[M+H]^+$	NA	3.04E-02
128.05284	C_6H_9NS	$[M+H]^+$	-0.089	3.11E-02
199.13281	$C_{11}H_{18}O_3$	$[M+H]^+$	-0.305	3.11E-02
131.06847	NA	$[M+H]^+$	NA	3.27E-02
119.95901	NA	$[M+H]^+$	NA	3.45E-02
69.05279	NA	$[M+H]^+$	NA	3.79E-02
149.09604	$C_{10}H_{12}O$	$[M+H]^+$	-0.352	3.84E-02
213.18491	$C_{13}H_{24}O_2$	$[M+H]^+$	0.025	3.91E-02
181.12214	$C_{11}H_{16}O_2$	$[M+H]^+$	-0.909	4.30E-02
125.05973	$C_7H_8O_2$	$[M+H]^+$	0.195	4.46E-02
122.08375	NA	$[M+H]^+$	NA	4.56E-02

m/z [a]	Molecular Formula (M)[b]	Adduct	Mass error (ppm)	p-value
151.07529	$C_9H_{10}O_2$	$[M+H]^+$	-0.432	4.60E-02
61.01061	C_2H_4S	$[M+H]^+$	-0.605	4.82E-02
120.10996	NA	$[M+H]^+$	NA	4.90E-02
96.05735	NA	$[M+H]^+$	NA	4.97E-02

[a] Exact mass (*m/z*) of the ion.

[b] Formula based on accurate mass (no isotopic pattern matching performed).

NA = not available.

Table S3. 22 features showing ^{13}C-incorporation after 24 h.

m/z[a]	Molecular Formula (M)[b]	Adduct	Mass error (ppm)
100.04741	NA	$[M+H]^+$	NA
101.05971	$C_5H_8O_2$	$[M+H]^+$	0.042
102.02667	NA	$[M+H]^+$	NA
102.06306	NA	$[M+H]^+$	NA
103.06641	NA	$[M+H]^+$	NA
104.03422	$C_3H_5O_3N$	$[M+H]^+$	0.006
105.00330	NA	$[M+H]^+$	NA
105.03688	C_4H_8OS	$[M+H]^+$	0.174
112.07569	C_6H_9ON	$[M+H]^+$	-0.002
117.06584	$C_4H_8O_2N_2$	$[M+H]^+$	-0.119
128.01645	C_5H_5ONS	$[M+H]^+$	-0.086
129.09100	$C_7H_{12}O_2$	$[M+H]^+$	-0.045
130.02321	NA	$[M+H]^+$	NA
130.09437	NA	$[M+H]^+$	NA
133.10431	$C_7H_{16}S$	$[M+H]^+$	-1.796
137.13247	$C_{10}H_{16}$	$[M+H]^+$	-0.048
138.13582	NA	$[M+H]^+$	NA
161.98717	NA	$[M+H]^+$	NA
169.15869	$C_{11}H_{20}O$	$[M+H]^+$	-0.007
170.16205	NA	$[M+H]^+$	NA
181.07176	$C_6H_{14}O_6$	$[M-H]^-$	-0.005
182.07512	$C_8H_{13}N_3S$	$[M-H]^-$	-3.392

[a] Exact mass (*m/z*) of the ion.

[b] Formula based on accurate mass (no isotopic pattern matching performed).

NA = not available.

Table S4. Top 25 features different between naïve and activated DCs.

m/z[a]	Molecular formula (M)[b]	Adduct	Mass error (ppm)	p-value	Fdr adj. p-value
117.03867	NA	$[M-H]^-$	NA	7.56E-07	2.77E-03
101.04832	NA	$[M+H]^+$	NA	7.30E-06	1.34E-02
148.04040	$C_8H_7O_2N$	$[M-H]^-$	-0.011	1.73E-05	1.87E-02
100.04741	NA	$[M+H]^+$	NA	2.04E-05	1.87E-02
128.01645	C_5H_5ONS	$[M+H]^+$	-0.086	6.26E-05	4.09E-02
237.01855	$C_7H_{12}ON_2S_3$	$[M+H]^+$	0.418	6.69E-05	4.09E-02
131.99365	$C_4H_5NS_2$	$[M+H]^+$	0.252	8.99E-05	4.71E-02
211.07540	$C_{14}H_{10}O_2$	$[M+H]^+$	0.210	1.11E-04	4.71E-02
233.21109	$C_{13}H_{28}O_3$	$[M+H]^+$	-0.131	1.16E-04	4.71E-02
134.04000	$C_3H_9ON_3S$	$[M-H]^-$	4.771	1.54E-04	5.65E-02
167.14421	NA	$[M+H]^+$	NA	1.70E-04	5.65E-02
161.08083	$C_7H_{12}O_4$	$[M+H]^+$	-0.031	2.51E-04	7.68E-02
162.93241	NA	$[M+H]^+$	NA	3.18E-04	8.96E-02
225.12739	$C_{16}H_{16}O$	$[M+H]^+$	-0.005	9.65E-04	2.53E-01
227.10666	$C_{15}H_{14}O_2$	$[M+H]^+$	0.019	1.50E-03	3.52E-01
225.09102	$C_{15}H_{12}O_2$	$[M+H]^+$	0.063	1.54E-03	3.52E-01
136.10493	NA	$[M+H]^+$	NA	1.76E-03	3.63E-01
116.04232	NA	$[M+H]^+$	NA	1.90E-03	3.63E-01
215.16527	$C_{12}H_{24}O_3$	$[M-H]^-$	0.013	1.97E-03	3.63E-01
179.09251	$C_7H_{16}O_5$	$[M-H]^-$	0.076	2.05E-03	3.63E-01
158.06004	$C_{10}H_7ON$	$[M+H]^+$	-0.001	2.09E-03	3.63E-01
160.06854	NA	$[M+H]^+$	NA	2.18E-03	3.63E-01
112.98804	$C_4H_2O_4$	$[M-H]^-$	0.072	2.44E-03	3.89E-01
256.26347	$C_{16}H_{33}ON$	$[M+H]^+$	-0.079	2.78E-03	4.24E-01
165.05957	NA	$[M+H]^+$	NA	3.29E-03	4.55E-01

[a] Exact mass (*m/z*) of the ion.

[b] Formula based on accurate mass (no isotopic pattern matching performed).

NA= not available.

Table S5. Top 29 altered metabolic pathways between naïve and activated DCs captured by real-time headspace analysis.

Pathway name	Total Size	Hits	Sig Hits	Mummichog Pvals	GSEA Pvals	Combined Pvals
alpha-Linolenic acid metabolism	12	1	1	1.39E-01	4.00E-02	3.45E-02
Valine, leucine and isoleucine degradation	35	12	5	1.52E-02	5.13E-01	4.56E-02
Citrate cycle (TCA cycle)	16	9	2	3.63E-01	3.85E-02	7.36E-02
Glycine, serine and threonine metabolism	31	16	4	1.67E-01	2.13E-01	1.54E-01
Valine, leucine and isoleucine biosynthesis	8	7	3	5.85E-02	6.33E-01	1.59E-01
Arginine and proline metabolism	37	21	4	3.31E-01	1.25E-01	1.73E-01
Alanine, aspartate and glutamate metabolism	28	15	3	3.48E-01	1.43E-01	1.99E-01
Propanoate metabolism	19	4	2	9.43E-02	6.11E-01	2.22E-01
Metabolism of xenobiotics by cytochrome P450	64	7	1	6.54E-01	1.01E-01	2.46E-01
Drug metabolism - other enzymes	27	5	2	1.44E-01	5.20E-01	2.68E-01
Pyruvate metabolism	19	4	1	4.53E-01	1.67E-01	2.71E-01
Phenylalanine, tyrosine and tryptophan biosynthesis	4	4	1	4.53E-01	1.81E-01	2.87E-01
Cysteine and methionine metabolism	33	11	1	8.14E-01	1.39E-01	3.60E-01
Tyrosine metabolism	42	34	6	3.27E-01	3.72E-01	3.78E-01
Drug metabolism - cytochrome P450	21	6	1	5.97E-01	2.15E-01	3.92E-01
Butanoate metabolism	15	8	2	3.08E-01	4.35E-01	4.03E-01
Phenylalanine metabolism	12	10	2	4.17E-01	3.46E-01	4.23E-01
Nicotinate and nicotinamide metabolism	15	7	2	2.52E-01	6.33E-01	4.53E-01
Pantothenate and CoA biosynthesis	17	7	2	2.52E-01	6.96E-01	4.81E-01
Vitamin B6 metabolism	9	4	1	4.53E-01	4.03E-01	4.93E-01
Terpenoid backbone biosynthesis	15	2	1	2.59E-01	7.21E-01	5.00E-01
Synthesis and degradation of ketone bodies	5	2	1	2.59E-01	9.15E-01	5.79E-01
Lysine degradation	19	9	1	7.46E-01	3.29E-01	5.90E-01
Glyoxylate and dicarboxylate metabolism	31	12	1	8.41E-01	4.55E-01	7.50E-01
Pyrimidine metabolism	39	10	1	7.83E-01	6.19E-01	8.36E-01
Pentose phosphate pathway	22	6	1	5.97E-01	9.11E-01	8.75E-01
Tryptophan metabolism	41	16	1	9.16E-01	6.82E-01	9.18E-01
Aminoacyl-tRNA biosynthesis	22	18	2	7.47E-01	9.04E-01	9.40E-01
Arginine biosynthesis	14	10	1	7.83E-01	8.64E-01	9.41E-01

References

1. Kind, T.; Fiehn, O., Seven Golden Rules for heuristic filtering of molecular formulas obtained by accurate mass spectrometry. *BMC Bioinformatics* **2007,** *8*, 105.
2. Keller, B. O.; Sui, J.; Young, A. B.; Whittal, R. M., Interferences and contaminants encountered in modern mass spectrometry. *Anal Chim Acta* **2008,** *627* (1), 71-81.
